# Mindfulness Awareness Practice (MAP) to Prevent Dementia in Older Adults with Mild Cognitive Impairment: Protocol of a Randomized Controlled Trial and Implementation Outcomes

**DOI:** 10.3390/ijerph181910205

**Published:** 2021-09-28

**Authors:** Ted Kheng Siang Ng, Lei Feng, Johnson Fam, Iris Rawtaer, Alan Prem Kumar, Grishma Rane, Irwin Kee-Mun Cheah, Ratha Mahendran, Yuan Kun Lee, Ene Choo Tan, Lee Gan Goh, Ee Heok Kua, Rathi Mahendran

**Affiliations:** 1 Department of Psychological Medicine, Yong Loo Lin School of Medicine, National University of Singapore, Singapore 117597, Singapore; Johnson_fam@nuhs.edu.sg (J.F.); pcmkeh@nus.edu.sg (E.H.K.); 2 Edson College of Nursing and Health Innovation, Arizona State University, Phoenix, AZ 85004, USA; 3 Institute of Behavioral Medicine, College of Medicine, National Cheng Kung University, Tainan 701, Taiwan; 4 Department of Psychological Medicine, National University Hospital, 1E Kent Ridge Road, Singapore 117599, Singapore; pcmfl@nus.edu.sg; 5 Department of Psychiatry, Sengkang General Hospital & SingHealth Duke-NUS Centre of Memory and Cognitive Disorders, Singapore 544886, Singapore; iris_rawtaer@hotmail.com; 6 Cancer Science Institute of Singapore, Yong Loo Lin School of Medicine, National University of Singapore, 28 Medical Drive, Singapore 117599, Singapore; apkumar@nus.edu.sg (A.P.K.); grishma.rane@nus.edu.sg (G.R.); 7 Department of Pharmacology, Yong Loo Lin School of Medicine, National University of Singapore, 28 Medical Drive, Singapore 117599, Singapore; 8 Department of Biochemistry, Yong Loo Lin School of Medicine, National University of Singapore, 8 Medical Drive, Singapore 117597, Singapore; bchickm@nus.edu.sg; 9 Department of Surgery, Yong Loo Lin School of Medicine, National University of Singapore, 1E Kent Ridge Road, Singapore 117599, Singapore; surrm@nus.edu.sg; 10 Department of Microbiology, Yong Loo Lin School of Medicine, National University of Singapore, 5 Science Drive 2, Singapore 117545, Singapore; micleeyk@nus.edu.sg; 11 Division of Clinical Support Services, KK Women’s and Children’s Hospital, 100 Bukit Timah Road, Singapore 229899, Singapore; Tan.Ene.Choo@kkh.com.sg; 12 Department of Family Medicine, National University Health System, 1E Kent Ridge Rd, Singapore 119228, Singapore; lee_gan_goh@nuhs.edu.sg

**Keywords:** mild cognitive impairment, aging, psychiatry, preclinical dementia, non-pharmacological intervention, mindfulness, health education, biomarker, neuroimaging

## Abstract

Background: With an aging population, developing non-pharmacological interventions (NPIs) to delay dementia has become critical. Apart from cognitive decline, dementia is associated with multiple pathophysiology, including increased oxidative stress, dysregulated gene expressions, cytokine, neurotrophin, and stress markers, telomere shortening, and deteriorations in brain connectivity. Although mindfulness practices have been proposed to ameliorate these biological changes, no empirical studies were conducted. We thus aimed to investigate the effects of mindfulness awareness practice (MAP) to prevent cognitive decline and improve peripheral biomarkers in community-dwelling older adults diagnosed with mild cognitive impairment (MCI). Methods/Design: This was a single-blinded and parallel-group randomized controlled trial with two arms (intervention and active control arms), conducted over nine months. A total of 60 consenting community-dwelling older adults diagnosed with MCI were planned to be randomized in a 1:1 ratio to either the MAP or the Health Education Program (HEP). Interventions were performed weekly for the initial 12 weeks, and monthly for the subsequent six months. Outcome measures were assessed at baseline, 3-month, and 9-month post-intervention by blinded assessors. Primary outcomes were neurocognitive tests, comprehensive peripheral biomarkers, and brain imaging scans. Secondary outcomes included basic health screening measures, affective symptoms, and measures of physical functions. Linear-mixed models were used to examine the effects of MAP on these outcome measures. Significance: This is the first randomized controlled trial to systematically investigate the effects of a mindfulness intervention in improving cognitive functions and various biomarkers in community-dwelling older adults diagnosed with MCI. Our findings have the potential to inform mindfulness intervention as a novel approach to delay dementia.

## 1. Introduction

With an accelerated demographic shift towards increased population aging, the incidence of dementia has been forecasted to escalate. In 2010, 35.6 million people were estimated to be diagnosed with dementia. It is postulated that this number will double every 20 years, culminating in 115.4 million cases in 2050 [1]. This issue has multi-faceted implications, including increasing burdens of affected patients, family members, caregivers, healthcare systems, and the economy. Dementia is, therefore, one of the most urgent public health issues globally. However, despite intensified efforts and numerous attempts in pharmaceutical trials, all new candidates for pharmacological intervention have failed in the past decade [2], except for one recent U.S. Food and Drug Administration (FDA)-approved drug. Furthermore, the existing treatments for dementia have limited efficacy and are only effective for a few months [3]. Hence, validating the potentials of preventive non-pharmaceutical interventions (NPIs) to slow and even reverse cognitive decline has received substantial attention in recent years, particularly at the preclinical dementia stage. Notably, if the onset and the progression of dementia could be delayed by just one year through any forms of interventions, there will be approximately 9.2 million fewer cases of dementia in 2050 [4], equaling an 8% decrease in the total projected dementia cases.

MCI is a transitional state between normal aging and very early dementia [5,6,7]. Individuals with MCI have an increased risk of dementia; although a small percentage of patients will revert to be cognitively healthy, 50% of MCI cases progress to develop AD [7]. Early identification, coupled with early preventive intervention at the MCI stage, could thus potentially slow and/or reverse cognitive decline. One way for such secondary prevention is through improving MCI- or dementia-associated modifiable risk factors [8]. Additionally, it has been previously shown that older adults with MCI are still cognitively able to learn and acquire new techniques [9]. With the pathology not as extensive as dementia, MCI could also potentially represent a period of malleable pathophysiology.

Given this immense potential, the search for non-pharmacological interventions (NPIs) to enhance cognition and to delay or even reverse cognitive decline has accelerated in the past decade. Amongst many different types of interventions, there have been increasing interests on the potential positive effects of mindfulness intervention on cognition. Mindfulness is defined as “paying attention in an intentional and non-judgmental way to the present moment” [10]. It has been shown to be a cost-effective, acceptable, and non-invasive approach to ameliorate the symptoms of a broad spectrum of disorders. Specifically, mindfulness interventions have demonstrated positive effects on various psychiatric disorders [11], including depression, social anxiety, obsessive-compulsive disorder, bipolar disorder, attention deficit disorder, and addiction. This raises the question of whether mindfulness interventions are applicable for older adults, who have comparatively short attention spans and a lifetime accumulation of allostatic stress [12]. Systematic reviews [13,14] concluded that meditation interventions, a closely related NPI, for older adults are feasible, with ample preliminary evidence suggesting that meditation may delay cognitive decline. However, most evidence was gleaned from epidemiological studies [15,16], many of which were of cross-sectional design, impeding causal inferences. Furthermore, the effectiveness of mindfulness intervention in older adults diagnosed with MCI is unknown.

Several theoretical propositions on how mindfulness could improve cognition have been postulated. Lutz et al. (2008) and McVay et al. (2009) proposed that by progressing through the two stages of mindfulness practices, namely focused attention and subsequently open monitoring, older adults’ attention and working memory could be improved [17,18]. Apart from attention and memory, executive function was also theorized to improve with mindfulness practice [19]. However, many prospective mindfulness interventional trials with non-MCI populations have shown conflicting results. To our best knowledge, only two small RCTs on mindfulness intervention with older adults diagnosed with MCI have been conducted, both with a small sample size of 14 participants each, and reported conflicting results in various cognitive measures [20,21]. This small sample size presents significant issues in achieving adequate statistical power, which could be one of the many reasons for the non-significant findings, instead of a lack of intervention effects [22]. Furthermore, a limited panel of outcome measures were collected in previous mindfulness RCTs with MCI, as they focused solely on cognitive outcomes and, to a smaller extent, neuroimaging measures [20,21]. Hence, there is a severe scarcity of evidence gathered from peripheral biomarker data.

The sole reliance on self-reported measures for assessing improvements upon undergoing mindfulness intervention has been identified as a major limitation [23,24,25,26]. This limitation calls for the need for biomarker examinations for mindfulness intervention [27]. As the pathophysiology of MCI and dementia is multi-faceted, they include the deterioration in neuronal functional connectivity, increased oxidative stress [28], increased low-grade systemic inflammation [29,30,31] and stress [32,33], decreased neurotropic factors [34], dysregulated gene expression [35], and shortening of telomere length [36]. However, no studies have yet to examine the effect of a mindfulness intervention on peripheral biomarkers in older adults clinically diagnosed with MCI. Hence, whether mindfulness intervention could significantly improve multiple biomarkers associated with MCI and dementia pathophysiology remains unknown. 

Several other significant methodological limitations could also be responsible for the mixed findings on the effects of mindfulness interventions. Amongst them, apart from limited causal inferences, epidemiological study design could also risk residual confounding. Even with the RCT design, some mindfulness interventions employed a single-group design and thus lacked a control group, which raises the possibility of bias and confounding. The 8-week intervention duration, which is typical of mindfulness interventions [37], raises questions on the long-term effects and the sustainability of the intervention. To the best of our knowledge, only one mindfulness trial has an extended follow-up period of up to 18 months, although the sample was not older adults diagnosed with MCI [38]. Furthermore, due to the inherent genetic and lifestyle differences, the effects of mindfulness intervention among Asian populations have remained mostly unexplored [39]. Conversely, we have previously demonstrated that mindfulness practice was acceptable to Singaporean Chinese and did not carry the stigma of mental illness [39], substantiating the rationales to perform such an intervention with Singaporeans. Because of these issues and motivations, a RCT on mindfulness intervention that addresses these limitations was advocated [40]. In this paper, we detail the protocol and implementation outcomes of this RCT. 

## 2. Aims

To address these gaps in knowledge, we conceptualized this parallel-group randomized control trial (RCT) to investigate the effects of a mindfulness intervention. We named it the Mindfulness Awareness Program (MAP), with community-dwelling Asian older adults clinically diagnosed with mild cognitive impairment, examining comprehensive outcome measures. We aimed to determine the effects of MAP compared to a Health Education Program (HEP), the active control arm, on (1) delaying or reversing cognitive decline amongst older adults diagnosed with MCI, (2) neuroimaging and comprehensive peripheral biomarkers associated with MCI, and (3) affective symptoms, namely depressive and anxiety symptoms, and physical functioning measures.

## 3. Methods and Design

### 3.1. Study Design and Setting

This trial is a parallel-arm and single-blinded RCT, which aimed to evaluate the effects of MAP over 9 months. The control arm partook in the HEP. The study protocol had been prospectively approved by the National University of Singapore Institutional Review Board (NUS IRB-Reference Code: B-14-110), and *a priori* registered at ClinicalTrials.gov (NCT02286791), a service of the United States National Institute of Health. We recruited our study participants from a larger on-going study, DaHA based at TaRA@JP [41]. It is a community-based research center established by the NUS Psychological Medicine department and located in a shopping mall, in the western part of Singapore. The primary recruitment strategy was through home visits to the government-built flats in public housing blocks located next to the study center. DaHA aimed to recruit older adults aged 60 and above. Once potential participants expressed interest to participate in the study, they were invited to the study center for informed consent taking, followed by detailed interviews and assessments. Dementia incidence was 4.6% at recruitment [42].

### 3.2. Study Participants—Eligibility Criteria

**Figure 1:**
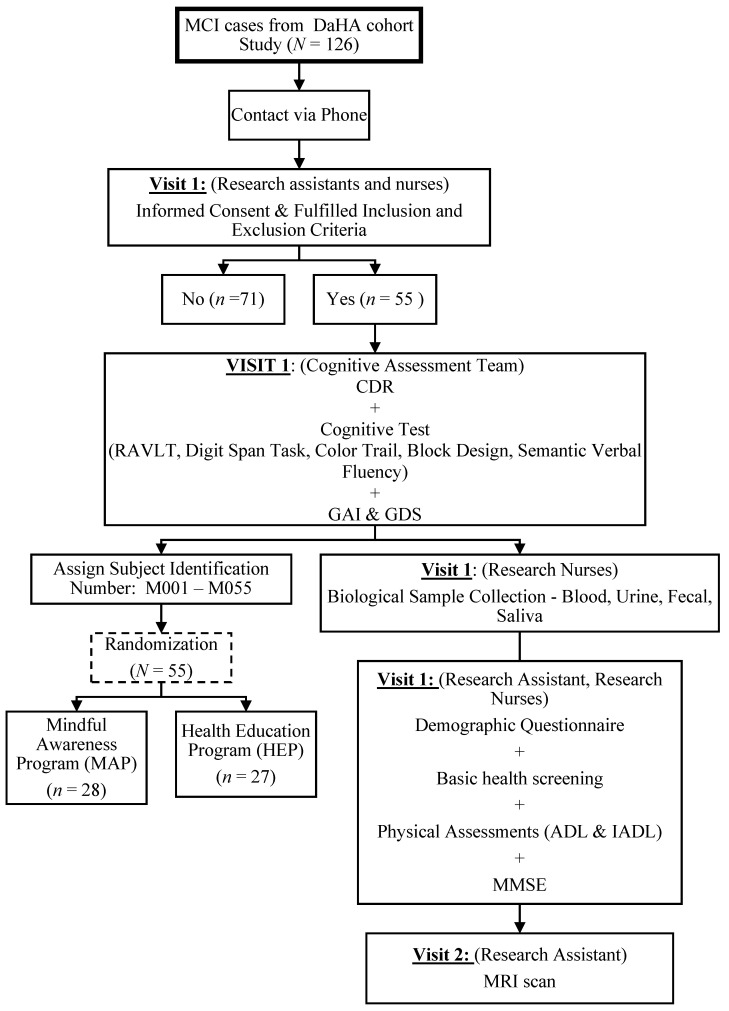
Protocol for Participant Screening, Recruitment, and Baseline Assessments. MCI = mild cognitive impairment; DaHA = Diet and Healthy Aging Study; CDR = clinical dementia rating; RAVLT = Rey Auditory Verbal Learning Test; GDS=Geriatric Depression Scale; GAI = Geriatric Anxiety Inventory; ADL = Activities of Daily Living; IADL = Instrumental Activities of Daily Living; MMSE = Mini-Mental State Examination; MRI = Magnetic Resonance Imaging.

The study was conducted in accordance with the Declaration of Helsinki. As shown in Figure 1, the participants were recruited from the Diet and Healthy Ageing Study (DaHA) (NUS-IRB Reference No:10-517) [41], a longitudinal cohort study based at the Training and Research Academy at Jurong Point (TaRA@JP). The research nurses, research assistants, and T.K.S.N. obtained written informed consent from the potential participants before screening for potentially eligible participants. All potential participants were screened for cognitive status and other eligibility criteria based on *a priori* inclusion and exclusion criteria.

The inclusion criteria were:

(1) Community-dwelling older adults aged between 60 and 85 years old and fulfilled the Peterson’s operational criteria of mild cognitive impairment [42]:
  - At least one age-education adjusted neurocognitive test Z-score < −1.5;
  - Do not meet DSM-IV criteria for major neurocognitive disorder;
  - Memory/cognitive complaint, corroborated by a reliable informant;
  - Intact activities of daily living.
(2) Function independently.
(3) Able to travel on their own to TaRA@JP and participate in the study.
(4) Does not have a diagnosis of dementia.

The exclusion criteria were:

(1) Dementia or normal ageing;
(2) Diagnosed with a neurological condition (e.g., epilepsy, Parkinson’s disease);
(3) Diagnosed with major psychiatric condition (e.g., major depressive disorder);
(4) Suffer from a terminal illness (e.g., cancer);
(5) Had significant visual or hearing impairment;
(6) Had marked upper and lower limb motor difficulties, which may affect their ability to participate in the study;
(7) Participation in another interventional study concurrently.

It is noteworthy that we did not exclude participants who did not consent to provide bio-specimen or MRI scans. In such cases, we continued with collecting all other measures to which the participants have consented, including interventions and neurocognitive outcome assessments. All participants were also newly-diagnosed community-dwelling MCI participants, who were naïve for medications targeting cognitive impairment at the start of the study.

### 3.3. Clinical Diagnostic Procedure for MCI

To derive the cognitive status of the participants, there was a two-step procedure. First, the assessors, comprised of a team of trained and certified research assistants and T.K.S.N., administered the clinical dementia rating (CDR) scale and neurocognitive assessments to all screened participants at the research center and derived preliminary research diagnoses. Subsequently, final research diagnoses of MCI were made during the study’s consensus meetings by a panel consisting of at least two senior consultant-ranked psychiatrists (E.H.K. and R.M.) and a clinical scientist (L.F.).

### 3.4. Recruitment and Baseline Procedures

**Figure 2:**
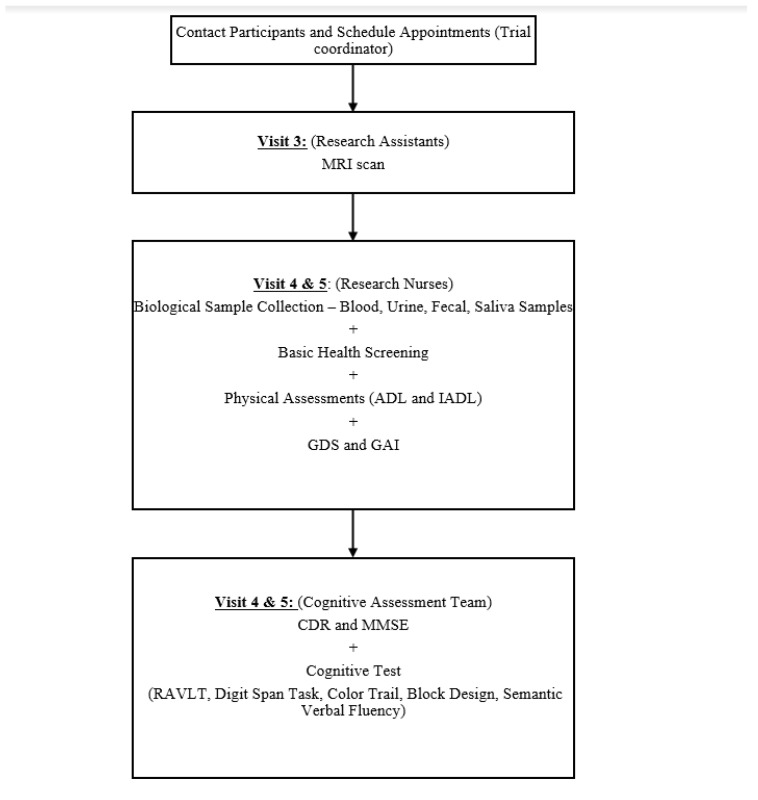
Flow Chart of the Follow-up Assessments at both 3-month and 9-month. MRI = Magnetic Resonance Imaging; ADL = Activities of Daily Living; IADL = Instrumental Activities of Daily Living; GDS = Geriatric Depression Scale; GAI = Geriatric Anxiety Inventory; CDR = Clinical Dementia Rating; RAVLT = Rey Auditory Verbal Learning Test; MMSE = Mini-Mental State Examination.

The participant flow from recruitment through to the end of study is shown in Figure 1 and Figure 2. Following the successful screening, baseline assessments were administered to all participants by trained research nurses and research assistants. The participants will then be randomized into one of the two study arms.

### 3.5. Randomization Procedure

After completing informed consent, screening, and baseline assessments, eligible participants were then randomized by an independent research assistant who was not affiliated with the trial. Using a random number generator function on the Random Allocation Software version 2.0 (Saghaei, Isfahan, Iran), the research assistant randomly allocated the participants in a 1:1 ratio to either the MAP or the HEP arm. The study’s research coordinator, F.N., then assigned the participants to the intervention arms. A single-blind design was employed; the assessors were blinded to the study arm assignments while the participants were aware of the study arms they were assigned to.

### 3.6. Delivery of Interventions

Since this is a parallel-arm RCT, the interventions for the two arms were conducted concurrently, at the same time of the same day and different rooms at the same research center. For the first three months, we held weekly intervention sessions for both arms, with each session of the MAP and HEP lasting approximately 1 h. For the study follow-up period, i.e., from 3-month to 9-month, 6 monthly booster sessions were held for both study arms. We recorded the attendance throughout the intervention period till the end of the 9-month period. We also provided the participants with personal diaries to record their practices at home. They were asked to return their session-specific diaries at the subsequent sessions to enable us to determine intervention adherence to daily practice and the frequencies of the practice. The sessions in each arm were structured with themes, led by the trainers with relevant experience and expertise.

### 3.7. Active Treatment Arm—Mindfulness Awareness Practice (MAP)

The treatment arm performed MAP, which was designed to foster awareness of internal states and promote positive mental and physical health through mindful awareness practices. MAP is a Singaporean version of the mindfulness intervention [43], modeled on the didactics of McBee mindfulness-based elder care (MBEC) [44], which adapted the mindfulness techniques to the unique needs of the older adult population [44]. We employed MBEC instead of mindfulness-based stress reduction (MBSR) due to several reasons; MBSR practices target the general population without focusing on the specific needs of older adults, including assuming that participants have good attention spans to understand and follow instructions and can commit to the practices and participate in simple exercises. However, older adults are often not able to follow many practices of the MBSR due to these requirements [45]. Thus, while maintaining the core intention of mindfulness, MBEC made adaptations to the MBSR model, making it tailored and uniquely suitable for older adults [45]. Each session was led by a certified and experienced instructor with years of experience teaching MAP techniques with the older adults. The various mindfulness techniques employed included:

(1) Mindfulness of the senses practice, which is to attend to the different senses (i.e., vision, hearing, touch) to cultivate focused attention. For example, the participants were requested to pay attention to their bodily senses while performing daily activities, including brushing their teeth.
(2) Mindful breathing with the body scan practice, where in a sitting position, participants were guided to develop kinesthesia by focusing their attention on various parts of their body as a relaxation technique. In more detail, the participants were guided by the instructor to place their attention on one part of their body at a time, while imagining directing their breath to the respective areas of their body and subsequently relaxing muscles at the specific areas. This practice was repeated, focusing on different body parts, starting from the toes to the head and vice versa.
(3) Movement nature meant a practice where participants were taught to move with awareness for flexibility, strength, and confidence. It was based on the Feldenkrais method aiming to restore natural coordination and movement mobility, incorporating two of the cornerstones of the Feldenkrais method, namely “Awareness Through Movement”^®^ and breathing awareness [46].
(4) Visuomotor coordination tasks, which trained the participants in mind-body coordination. The participants were guided to perform different cognitively demanding tasks requiring the fine coordination of the visual and motor systems. For example, the participants used one hand to perform a task with rubber bands and then switched quickly to performing the same task using the other hand, back and forth to be repeated a few times.
(5) Lastly, mindful stretching aimed to relax the muscles, by requiring the participants to stand up and stretch their muscles mindfully, while sighing and releasing the tensions felt in the muscles.

**Figure 3:**
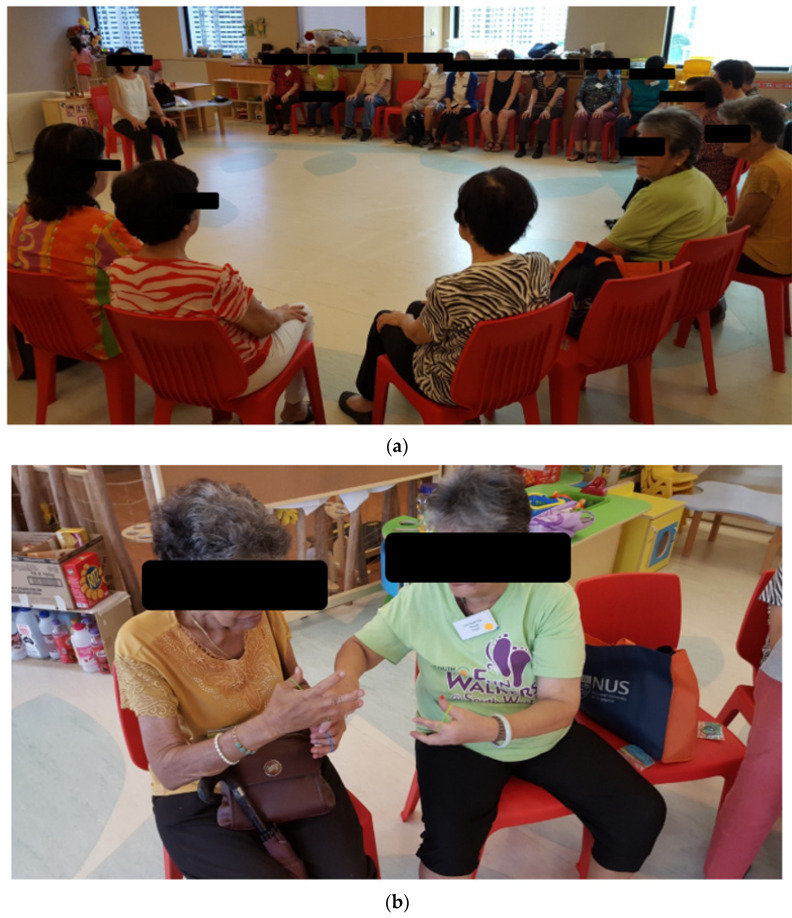
(**a**,**b**) Examples of MAP intervention sessions (top: mindful breathing, bottom: visual-motor coordination task).

Figure 3a,b shows the pictures of the participants practicing the mindfulness practice during the intervention sessions.

### 3.8. Active Control Arm—Health Education Program (HEP)

Modeled on the HEP proposed by MacCoon et al. [47], the active comparator group participated in a series of talks on health-related topics of interest to older adults. The weekly topics covered hypertension, diabetes, dementia, depression, medications, exercise, diet, sleep, home safety, falls and social support. The monthly sessions reviewed the topics and discussed how the participants had implemented healthy practices in their daily lives. Due to the breadth of the topics covered, the program was delivered by a panel of healthcare professionals specialized in the topics, which included clinicians, nurses, and psychologists. It was recommended to employ health education as the active control arm in mind-body intervention, to control for components that are non-intervention-specific [48].

### 3.9. Data Management

The trial coordinator monitored the screening and recruitment procedures to ensure the informed consent process was being properly conducted and obtained prior to the start of the study, in line with the institutional IRB. Upon successful screening, informed consent and enrollment, each participant was assigned a unique numerical study number with all the subsequent documents and sample labeling using the same study number. Only the Principal Investigator (R.M.) and the study coordinator have access to the identifiers. Data (Table 1) were entered and stored on a standard desktop computer with password protection. Following the NUS data management policy (DPRT-2011-04), all data were prospectively kept for ten years, beyond which they will be destroyed. Data entry and processing procedures ensured that the blinding was maintained during the entire course of trial. A backup system was in place; apart from the files stored at the desktop at the research center, i.e., TaRA@JP, another copy of all the files were stored in an external hard drive.

Two databases were created: (1) an administrative database, a Microsoft Excel spreadsheet to compile data related to administrative matters and documentation process. They included participant identification data, administrative data, and regulatory data, and (2) research database, which included a compilation of data that were used for documenting outcome measures and statistical analyses. For both databases, they were secured and encrypted with password protection, with access granted only to the Principal Investigator, the study coordinator, and a data entry staff member. The primary and secondary measures were independently reviewed by a research assistant to ensure correct data entry.

Any hardcopy data collection forms transferred from TaRA@JP to the office at National University Health System were sent via courier or registered post, or transported by taxi or private transport, to minimize the risk of data loss. The consent forms and data collection forms were stored in a locked cabinet at TaRA@JP, and access to them was only made available by the Principal Investigator or the study coordinator.

### 3.10. Statistical Analysis

Based on previous studies [35,49,50,51] examining the effects of mindfulness on the means and SDs for co-primary outcomes chosen for this study, i.e., fluid biomarkers and neurocognitive tests, we estimated the effect sizes to be 0.5. Hence, we required 24 participants in each group to have a power of 80% to detect statistical significance at 5% level. Considering a potential 20% drop-out rate, 30 participants needed to be assigned to each group at baseline. Hence, the targeted total sample size was 60.

All demographic and outcome measures were expressed as mean ± standard deviation (SD). We used either Fisher’s exact, Student’s or chi-square *t*-test to examine the differences in the baseline variables, as per the nature of the data. In the event the raw values of the measures were not conforming to the normality assumption, the raw values were natural log-transformed for subsequent analyses. Successful normalizations were based on dot plots, skewness, and kurtosis.

We employed the linear-mixed model to examine the treatment effects of MAP on the biomarkers and psychometric scale outcome measures, compared to the active control group, HEP, over the three time-points. For the psychometric and fluid biomarker measures, we performed intention-to-treat analyses using linear-mixed model, because it is robust to missing data. The model considers all the participants included in the baseline, regardless of whether they have missing values at subsequent follow-ups [52]. For the neuroimaging measures, since the drop-out rates were much higher, we used per-protocol analyses, where only participants that completed the intervention and had neurocognitive and neuroimaging scans at follow-ups were included in the ANOVA analyses.

In each of the linear-mixed models, we entered the outcome measure of interest as the dependent variable. The time-points of the intervention, treatment arm, and an interaction term between time-points and treatment arm were treated as the independent variables. Covariates for all the models included the baseline values of the respective outcome variable, age, sex, and years of formal education. We also collected measures for other covariates relevant to MCI and dementia, including the geriatric depression scale (GDS) and geriatric anxiety inventory (GAI) clinical cut-offs, history of cardiovascular diseases, and history of myocardial infarction. These additional covariates were explored to examine their effects on the basic models, using the model fit statistics, namely the Akaike information criterion (AIC) and Bayesian information criterion (BIC) values. In the final models, for the additional covariates, only the significant ones for each model were retained. We based all the analyses on the intention-to-treat principle, hence all the participants who had measures at at least one time-point were included in the analysis. All statistical analyses were and will be further performed using the Statistical Package for the Social Sciences (SPSS) Statistics for Windows, version 23.0 (IBM Corp., Armonk, NY, USA). A two-tailed *p*-value of < 0.05 was and will be considered statistically significant.

## 4. Results

### 4.1. Reach (Recruitment): Screening and Recruitment Rate

As shown in Figure 1, from the larger DaHA cohort, we identified N = 126 potential MCI cases. They were contacted by phone to be briefed on the study and to sign informed consent in person. Upon screening using the neurocognitive tests, of the 126 potential participants, 71 were excluded. Of the 71, 25 did not fulfill the inclusion and exclusion criteria, whereas 46 declined to participate. A total of 55 signed informed consent (recruitment rate = 43.65%) and were subsequently randomized into either the MAP or the HEP groups.

### 4.2. Implementation (Randomization Outcome): Balance in Baseline Variables

There were no significant differences in all baseline variables, including age, sex, and education levels (Table 2). All randomized participants attended the intervention groups that they were initially randomized to.

### 4.3. Maintenance: Adherence Rate and Lost to Follow-Up for Primary and Secondary Outcome Measures

**Figure 4:**
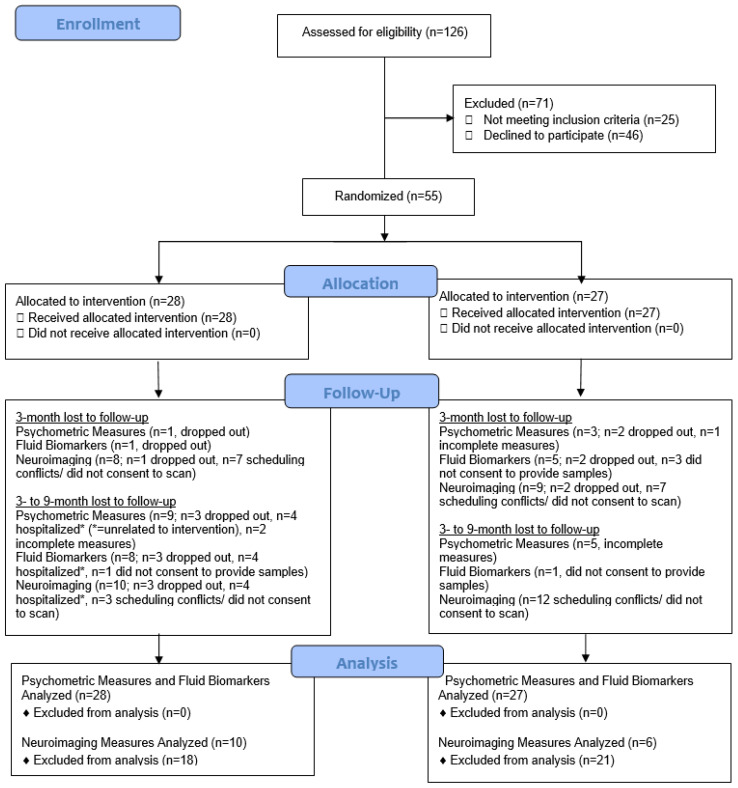
CONSORT 2010 Flow diagram for co-primary outcome measures.

Adherence or attendance rates were approximately 88 and 89% for the MAP and HEP groups, respectively. Since we did not require all the participants to consent to bio-specimen collection and neuroimaging scans, there could be differences in lost to follow-ups. Lost to follow-ups for fluid biomarker, psychometric and neuroimaging measures are presented in Figure 4.

For the fluid biomarker measures, the total number of lost to follow-up cases at 9-month time-point was 9 in the MAP (32.14%) and 6 (22.22%) in the HEP group. For the psychometric measures, similar lost to follow-up rates at 9-month time-point were observed: 10 in the MAP (35.71%) and 8 in the HEP group (29.63%). For the neuroimaging measures, there were 18 lost to follow up cases in the MAP (64.29%) and 21 lost to follow up cases in the HEP group (77.78%).

## 5. Discussion

Despite apparent differences in genetics and lifestyle factors, most mindfulness intervention trials were conducted in the Western Hemisphere. The effects of a mindfulness intervention among Asian populations remained largely unexplored [39]. We previously showed that mindfulness practice was acceptable to Singaporean Chinese [43,53]. Furthermore, it did not carry the stigma of mental illness, unlike other psychotherapeutic approaches. Taken together, no mindfulness intervention focusing on cognition and peripheral biomarkers in older adults with MCI, utilizing parallel-group RCT design, has been conducted with an Asian population. This gap in knowledge motivated this trial.

This randomized controlled trial was designed to holistically test the bio-psychological effects of the mindful awareness program for older adults diagnosed with MCI. Extant NPIs targeting the traditional lifestyle factors, such as diets and regular exercise, have shown mixed evidence [54,55,56]. Despite that cognitive training programs, including mindfulness interventions, have been shown to slow cognitive decline in cognitively healthy older adults, evidence on preventing or delaying cognitive decline in MCI, who are older adults at greater risk of developing dementia, is in great scarcity [57]. Targeting this vulnerable group of older adults is imperative, as amongst older adults diagnosed with MCI, despite studies showing a significant decrease in quality of life and functions in various cognitive domains [58] and the existence of modifiable risk factors, they have not been the focus population for NPIs. Two previous RCTs on MCI also present several pertinent methodological issues, warranting future investigations.

This RCT thus filled the gap in the literature for a robust and rigorously conducted RCT with the inclusion of an active control group to determine the effectiveness of a mindfulness intervention with older adults clinically diagnosed with MCI [51]. In particular, this study contributes to addressing the scarcity in the literature on mindfulness intervention focusing on an array of multiple cognitive abilities and diverse peripheral biomarkers in older adults with MCI, utilizing parallel-group RCT design, which is conducted with an Asian population. The neurocognitive tests, measures on affective symptoms and physical limitations, neuroimaging scans, a comprehensive set of peripheral biomarkers encompassing blood, saliva, fecal, and urine biomarkers provided pilot evidence of the biological effects and thus illuminated the biological underpinnings of mindfulness intervention with older adults diagnosed with MCI. This RCT is, to date, the largest RCT on mindfulness intervention with older adults clinically diagnosed with MCI. Furthermore, this study did not only study the short-term effects, but also the long-term effects of mindfulness intervention, making this study one of the longest interventional trials employing mindfulness as a novel approach to delay cognitive impairments and studying its long-term cognitive and biological effects systematically.

Several implementation outcomes are worth noting. First, the study fell short of the targeted 60 participants and recruited 55 participants instead. Coupled with the higher-than-expected drop-out rates, the power of the trial was lower than the calculated 80%. In this parallel-group trial, the participants in the HEP group attended the intervention at the same time and same day throughout the intervention period as MAP, but separately and in different rooms at our research center. Hence, there were minimal interactions. However, we acknowledged that there was still plausible inadvertent contamination due to the participants conversing and interacting before and after their respective intervention sessions. One mitigation was that we let the participants in the MAP arm leave the intervention site, i.e., our research center, earlier than the HEP participants, minimizing the contamination. The registration booths were also separate for the two groups. The recruitment rate, 43.65% (55 recruited out of 126 screened), is lower than another RCT targeting participants with MCI, 66.81% [59]. Conversely, the attendance and retention rates were approximately 88 and 89%. These figures are very similar to other psychosocial interventions targeting MCI, which are approximately 90 and 85%, respectively [20,60]. We strengthened follow-ups and adherence and minimized potential drop-outs by having the study’s coordinator and research assistants conduct weekly reminder calls to the participants or family members three days prior to each intervention session. Since our target sample population had cognitive impairments, these regular reminder calls could have ensured high adherence rates to the interventions. The instructors played a critical role in both the MAP and HEP. Compared to conducting interventions on cognitively intact older adults, enhanced instructors’ interaction was needed to effectively and continuously engage this population. Enhanced interaction with the instructors might explain the high attendance rate as well. We were unable to accurately determine the adherence rates of the interventions. Although we provided homework and personal diaries to the participants at the end of each session, the participants often did not bring their personal diaries back to the research center for fidelity check purpose. Based on the feedback from the instructors and our interaction with the participants, we suspect that due to having MCI, many participants did not perform the home practices assigned. For future studies, due to the on-going COVID-19 pandemic, one future direction is to use mobile health technology, while considering the electronic literacy of older adults. Other implementation strategies could be found in our previous paper [61]. The lost to follow-up rates for fluid biomarkers and neurocognitive tests were very similar, although for the neuroimaging measures, the rates were high in both the MAP and HEP groups. By delineating the implementation outcomes and lessons learned from this pilot mindfulness trial with MCI, we hope they will serve as a guide for future study design and implementations. Lastly, no adverse events related to either the MAP or HEP intervention was reported, suggesting the feasibility and safety of the interventions.

This trial has several limitations. Despite the encouraging findings, this is a proof-of-concept study. With encouraging findings, future validation in larger RCTs is needed. Blinding of the participants to the assigned interventions was not feasible. One potential mitigation is to use sham meditation as the active control arm [37]. Due to limitations in logistics and human resources, only a single time-point sample of saliva samples were collected for each participant at each time-point. Thus, the findings for salivary cortisol levels reported should be interpreted with caution. For higher accuracy and reliability, the ideal saliva sampling method is to collect saliva samples at multiple time-points throughout the day and take the reading from the area under the curve or the concentration versus time curve with respect to zero [62]. Lastly, the absence of significant *p*-values for several outcome measures might have been attributed to the study’s slightly reduced power, as the original recruitment target was not met.

Despite the limitations, this study represents a significant advancement on several fronts. First, we employed an RCT design, with the active control arm controlled for several intervention components, which were not specific to mindfulness. Adding the active control group proposed by MacCoon et al. (2012) [47] thus considerably improved the study’s conclusions on effectiveness. This RCT included equal instructor’s attention for both intervention groups [37], and interventions were conducted at the same time of the day and on the same day of the week [23], and had equal length of the sessions [37]. This study design minimized residual confounding effects, by ensuring that the two study arms differed mainly in the interventions being compared [63]; this is the first RCT to concomitantly and comprehensively examine a range of psychometric scales and biomarkers representing different biological mechanisms, allowing us to pinpoint the specific effects of MAP in MCI, and contributing to filling the dearth of knowledge of mindfulness intervention on biomarkers in MCI. This study also addressed one of the main limitations present in the mindfulness intervention literature, i.e., the 8-week short follow-up period, by the addition of monthly booster sessions from 3- to 9-month. Lastly, to our knowledge, although with moderate power, compared to previous two RCTs conducted with MCI (N = 14) [20,21], this study is to date the largest and longest follow-up RCT of mindfulness intervention conducted with older adults diagnosed with MCI. With this extended follow-up period, we elucidated the long-term effects of mindfulness, contributing to expanding the knowledge on this topic.

## 6. Conclusions

In investigating the multi-faceted biopsychological outcomes of MCI, this study presented pilot data on whether mindfulness intervention improved cognition and several well-established pathophysiology of MCI and dementia. As we have shown, the effectiveness of a mindfulness intervention with MCI with the use of an active control group, future studies could add an additional wait-list control group to further evaluate the efficacy of the intervention. Apart from showing pilot data on MCI as an intermediate disease stage that is amenable to mindfulness psychologically, positive findings on the biomarkers also opened the possibility that MCI represents a stage of disease with malleable biology. Hence, this RCT presented actionable targets, which are modifiable risk factors of dementia, in response to a novel mindfulness intervention. The results from this RCT could inform policymakers of the need to support low-cost and self-directed psycho-social programs and activities to prevent dementia and even reverse cognitive decline in MCI. Indeed, this RCT, together with the other RCTs involving cognitive stimulating activities we have previously conducted [43,64,65,66,67], provided pilot scientific evidence for the nationwide rollout of the dementia prevention program, termed the Aging-Well-Everyday (AWE) program, in Singapore. Backed by robust scientific evidence, this is a first-of-its-kind community-based multimodal intervention performed by fostering close collaboration among governmental organizations, non-governmental organizations, and academic institutions, enabling a truly community-integrated model. For sustainability of the AWE program, we work closely with community centers in eight housing estates, and these centers are partly funded by the People’s Association which has financial support from the government. With our online program in the Malay language, we hope to extend to neighboring countries, such as Indonesia and Malaysia. We envision that this could be the model for future studies on novel NPIs to examine real-world intervention effects, to extend the interventions’ benefits to the community and potentially halt the increased incidence of dementia. With mindfulness intervention as a health promotion and secondary preventive strategy, older adults could age in place in the community.

### Trial Status

The study has completed intervention and data collection. Part of the data analyses have been completed and published [61,65,68,69,70]. The remaining analyses are estimated to be completed by December 2021. Although several recent publications describing the results of this trial have been published, this paper has its distinct scientific contribution in detailing the step-by-step procedures of the trial. As the first RCT on mindfulness intervention with older adults diagnosed with MCI that incorporated comprehensive biomarker measures as objective outcome measures, this study could inform future study designs and facilitate replication studies in advancing mindfulness intervention targeting older adults with MCI.

## Figures and Tables

**Table 1:** A summary of assessments or measures collected throughout the three time-points.

Assessments or Measures	Baseline	At 3-Month	At 9-Month
Social-demographic Questionnaire	√	-	-
Basic Health Screening	√	√	√
Physical Assessments (ADL and IADL)	√	√	√
Mini Mental State Examination (MMSE)	√	√	√
Depression and Anxiety Screening Tests (GDS and GAI)	√	√	√
Clinical Dementia Rating (CDR) Scale	√	√	√
Neurocognitive Tests	√	√	√
MRI Brain Scan	√	√	-
Blood Sample	13 mL	8 mL	13 mL
Urine Sample	15 mL	-	15 mL
Fecal Sample	√	√	√
Saliva Sample	√	√	√

ADL = Activities of Daily Living; IADL = Instrumental Activities of Daily Living; MMSE = Mini-Mental State Examination; GDS = Geriatric Depression Scale; GAI = Geriatric Anxiety Inventory; CDR = clinical dementia rating; MRI = Magnetic Resonance Imaging.

**Table 2:** Comparisons of the baseline demographic and characteristics between participants in the Mindful Awareness Practice (MAP) and Health Education Program (HEP) Arms (N = 55).

Baseline Demographics and Characteristics	MAP, Treatment; n (%) or Mean (SE) (n = 28)	HEP, Control; n (%) or Mean (SE) (n = 27)	*p*-Value
Age (in years)	71.89 (1.14)	70.67 (1.19)	0.46
Sex			
Male	8 (28.60%)	6 (22.20%)	0.59
Female	20 (71.40%)	21 (77.80%)	
MCI subtypes			
aMCI	13 (46.40%)	8 (29.6%)	0.27
Non-aMCI	15 (53.6%)	19 (70.4%)	
Years of Formal Education	5.27 (5.02)	3.44 (4.27)	0.16
Ethnicity			
Chinese	27 (96.40%)	27 (100%)	1.00
Indian	1 (3.60%)	0 (0%)	
Others	0 (0%)	0 (0%)	
Employment Status			
Retired	14 (51.90%)	11 (40.70%)	0.14
Full-time worker	0 (0%)	0 (0%)	
Part-time worker	0 (0%)	4 (14.80%)	
Housewife	13 (48.10%)	12 (44.40%)	
Marital Status			
Single	1 (3.70%)	0 (0%)	0.29
Married	18 (66.70%)	14 (51.90%)	
Divorced	2 (7.40%)	1 (3.70%)	
Widowed	6 (22.20%)	12 (44.40%)	
MMSE (Total Scores)	24.59 (0.63)	24.70 (0.75)	0.91
GDS			
<5	18 (64.30%)	22 (81.50%)	0.15
≥5	10 (35.70%)	5 (18.50%)	
Attendance rate (%)	88.6 (12.48)	87.0 (19.11)	0.77
Total number of metabolic disorders	1.44 (0.22)	1.52 (0.16)	0.79
Presence of diabetes	6 (22.2%)	8 (29.6%)	0.76
BMI, kg/m^2^	24.76 (0.85)	24.06 (0.67)	0.53
Total number of chronic diseases	2.04 (0.33)	2.85 (0.25)	0.66
Total number of medications taken	2.89 (0.44)	2.96 (0.39)	0.90
Total number of participants taking psychotropic medications	1 (3.70%)	0 (0%)	1.00

Abbreviations: aMCI = amnestic MCI; non-aMCI = non-amnestic MCI; BMI, body mass index; MMSE, Mini-Mental State Examination; GDS, Geriatric Depression Scale; clinical cut-off for GDS is 5, with those ≥5 suggesting probable depression.

## Data Availability

Data may be available from the corresponding authors upon reasonable requests.

## Abbreviations

ADL: Activities of Daily Living; CDR: Clinical Dementia Rating; COPs: cholesterol oxidation products; DTI: Diffusion Tractography Imaging; ELISA: enzyme-linked immunosorbent assays; fMRI: Functional Magnetic Resonance Imaging; GAI: Geriatric Anxiety Inventory; GDS: Geriatric Depression Scale; HEP: Health Education Program; IADL: Instrumental Activities of Daily Living; MAP: Mindful Awareness Program; MAP-RCT: Mindful Awareness Program Randomized Controlled Trial; MCI: Mild Cognitive Impairment; MMSE: Mini-Mental State Examination; SD: standard deviation; TaRA@JP: Training and Research Academy at Jurong Point.
